# Resting-state functional connectivity of salience network in schizophrenia and depression

**DOI:** 10.1038/s41598-022-15489-9

**Published:** 2022-07-01

**Authors:** Huan Huang, Cheng Chen, Bei Rong, Qirong Wan, Jingang Chen, Zhongchun Liu, Yuan Zhou, Gaohua Wang, Huiling Wang

**Affiliations:** 1 Department of Psychiatry, Renmin Hospital of Wuhan University, Wuhan, 430060 China; 2 Department of Psychiatry, Zhongxiang Hospital of Renmin Hospital of Wuhan University, Zhongxiang, 431900 China; 3 Institute of Psychology, Chinese Academy of Sciences, Beijing, 100101 China; 4 Hubei Institute of Neurology and Psychiatry Research, Wuhan, 430060 China; 5 Hubei Provincial Key Laboratory of Developmentally Originated Disease, Wuhan, 430071 China

**Keywords:** Depression, Schizophrenia, Neuroscience

## Abstract

To explore the salience network (SN) functional alterations in schizophrenia and depression, resting-state functional magnetic resonance imaging (rs-fMRI) data from 29 patients with schizophrenia (SCH), 28 patients with depression (DEP) and 30 healthy controls (HC) were obtained. The SN was derived from data-driven group independent component analysis (gICA). ANCOVA and post hoc tests were performed to discover the FC differences of SN between groups. The ANCOVA demonstrated a significant group effect in FC with right inferior and middle temporal gyrus (ITG and MTG), left caudate, and right precentral gyrus. Post-hoc analyses revealed an opposite altered FC pattern between SN and right ITG and MTG for both patient groups. The DEP group showed a reduced FC between SN and right ITG and MTG compared with HC whereas the SCH group showed an increased FC. In addition, the SCH group showed decreased FC between SN and left caudate, and enhanced FC between SN and right precentral gyrus compared to the other two groups. Our findings suggest distinct FC of SN in schizophrenia and depression, supporting that the resting-state FC pattern of SN may be a transdiagnostic difference between depression and schizophrenia and may play a critical role in the pathogenesis of these two disorders.

## Introduction

Schizophrenia and depression are two of the three most common serious psychiatric disorders. Considerable overlap in symptomatology and clinical features such as emotional and cognitive dysregulation could be seen in both diseases^1^, and genetic studies have also identified some common polymorphisms^2^. All these findings suggest that they may share neurobiological commonalities, as well as disease-specific mechanisms.

In recent years, with rapid development of neuroimaging techniques, researchers have widely explored the brain in people with mental disorders, and various structural and functional changes have been associated with both disorders. Among these, resting state functional magnetic resonance imaging (rs-fMRI) has been widely used to explore the biological markers in psychiatry, as they are relatively simple and easy to acquire, particularly in patients with mental disorders^3,4^. Rs-fMRI is a main research method in this field and the functional connectivity (FC) is the one of most common analytic strategies, which is defined as a statistical temporal correlation of low frequency fluctuations between anatomically distinct brain regions^5^. Previous evidence has illustrated that both schizophrenia and depression have been generally identified in relation to the aberrant functioning and organization of large-scale resting state networks (RSNs)^3,6–8^. RSNs are defined by the specific FC of ongoing, slowly fluctuating brain activity, including typical default mode network (DMN), the central executive network (CEN), and the salience network (SN), etc^9^.

Until now, relatively few rs-fMRI studies have been conducted to directly compare patients with schizophrenia and depression and the results are inconsistent. Schilbach et al. assessed transdiagnostic aberrations of resting state FC of DMN in schizophrenia and patients with depression from patients from multiple study centers using seed-based resting state analyses and their results demonstrated a common decrease of FC between precuneus and bilateral superior parietal lobe in both disorders, and further identified the reduced FC between DMN and parietal operculum as the diagnosis-specific feature in schizophrenia relative to depression^10^. Resting-state FC pattern in the prefrontal cortex may been regarded as a potential transdiagnostic difference between depression and schizophrenia patients^11^. Jiang et al. found both common and distinct structural deficits and aberrant causal connectivity patterns in the prefrontal–thalamic–cerebellar circuit in schizophrenia and depression^12^. In addition, recent studies support that SN abnormality may play a critical role in the pathogenesis of schizophrenia and depression^13,14^. The SN comprises cortical and subcortical prominent nodes in the anterior cingulate cortex (dACC), as well as the rostral prefrontal cortex (RPFC) and the supramarginal gyrus (SMG). The SN also includes subcortical limbic areas including the striatum, thalamus and amygdala, which have been identified in fMRI studies^15–20^. The function of the SN might be to identify the most homeostatically relevant among multiple internal and external stimuli that constantly accost the brain, and is commonly coactivated across a wide range of cognitive and affective tasks^17,21^. Moreover, researchers have found that SN manifested the highest levels of time-varying flexibility in connectivity with other networks and suggested the SN is a unique hub for driving inter-network interactions and acts as a “dynamic switch”^19^.

However, the FC abnormalities in SN between the two disorders are still unclear. The aim of the study is to directly compare the FC of SN in both disorders and identify common and different FC patterns to explore the pathophysiological mechanism underlying aberrant SN connectivity in schizophrenia and depression. We speculated that aberrant functional connectivity patterns of SN exist in patients with schizophrenia and depression. In this study, we used rs-fMRI to investigate FC patterns of SN in patients with schizophrenia, depression, and a matched group of health controls. We defined the SN from the data-driven group independent component analysis (gICA), and further explored the FC between the SN and the whole brain.

## Materials and methods

### Participants

This study recruited 30 individuals with schizophrenia (SCH group), 30 individuals with depression (DEP group), and 30 healthy controls (HC group). The participants from three groups were matched for age, gender and years of education. All patients were recruited from the inpatients and outpatients at the department of psychiatry, Renmin Hospital of Wuhan University. The diagnose of schizophrenia or major depression disorder was made by two clinical psychiatrists independently according to the DSM-IV Disorder-Clinical Version (SCID-CV). Symptom severity was assessed using the Positive and Negative Syndrome Scale (PANSS) for schizophrenia patients and the 17-items Hamilton Depression Rating Scale (HAMD-17) for patients with depression. All of the patients were in an acute phase. For the first-episode patients, they have never taken any antidepressants or antipsychotics before being recruited in this study; for the multiple-episode patients, they discontinued their psychotropic drug treatment for more than 6 months. However, all patients were medicated following established medication regimes by their psychiatrist for less than one week. The healthy participants were recruited from the hospital faculty and local community via advertisement. They had neither lifetime psychiatric disorder nor family history of psychosis in their first-degree relatives.

Participants in the three groups were excluded if they had any neurologic disorder (including cerebrovascular disease, neoplasms, trauma, infection, degeneration and epilepsy), other concomitant major physical illnesses, dementia, brain injuries, substance abuse or addiction, or contraindications to MRI. Three participants were excluded from data analyses due to artefacts, leaving 30 healthy controls, 28 patients with depression and 29 patients with schizophrenia to be included in the analyses.

This study was approved by the Ethics Committee of Renmin Hospital of Wuhan University, and all participants provided their written informed consent individually after the study was completely described to each participant. In addition, we confirm that all methods were carried out in accordance with relevant guidelines and regulations.

### Imaging data acquisition and preprocessing

Whole-brain imaging data were performed on a 3.0T General Electric (GE) Signa HDxt MR scanner at the department of radiology, Renmin Hospital of Wuhan University. The subjects were instructed to keep their eyes closed without falling asleep, relax, and not to focus their thoughts on anything in particular.

Functional images were collected using a gradient echo planar imaging (EPI) sequence with following main scan parameters: TR = 2000 ms; TE = 30 ms; flip angle = 90°; FOV = 220 mm × 220 mm; matrix = 64 × 64; slice = 32; slice thickness = 4 mm; gap = 0.6 mm. Each functional scan session lasted 8 min, resulting in 240 volumes to be obtained. In addition, high-resolution T1-weighted structural images were obtained using a magnetization-prepared rapid acquisition gradient echo sequence (TE = 7.8 ms; TR = 3.0 ms; flip angle = 7°; inversion time = 1100 ms; FOV = 256 mm × 256 mm; matrix = 256 × 256; slice = 188; voxel size = 1 mm × 1 mm × 1 mm).

The functional data preprocessing was performed in MATLAB using the CONN toolbox 19c (https://www.nitrc.org/projects/conn) and SPM12 (http://www.fil.ion.ucl.ac.uk/spm/software/spm12/). All functional images underwent the default preprocessing pipeline for volume-based analyses^22^, which consisted of (1) removing the first five volumes to allow the subjects’ adaptation to the scanning environment and make signal equilibrium, (2) functional realignment, (3) functional slice-timing correction,(4) functional outliers detection (ART-based identification of outliers scans for scrubbing^23^), (5) functional direct segmentation and normalization to Montreal Neurological Institute (MNI) space, (6) structural segmentation and normalization to MNI space, (7) functional smoothing using a Gaussian kernel with full-width at half-maximum of 6 mm. For the scrubbing, outliers were defined as composite movement greater than 2 mm or more than 3 standard deviations away from the mean image intensity, individuals were excluded if > 20% of time points were identified as outliers; however, no participants met this exclusion criterion. Then in the denoising step, all data were default band-pass filtered (0.008–0.09 Hz), and the white matter and cerebrospinal fluid were regressed out and head motion related artifacts were reduced using the anatomical component-based noise correction (CompCor) strategy^24^.

### Independent component analysis

The ICA was performed on all participants together. Using default settings for CONN 19c, the data-driven ICA was run with a G1 FastICA for estimation of independent spatial components and GICA3 back-projection for individual subject level spatial map estimation. The default recommended value for dimensionality reduction in CONN toolbox keeps the first 64 components to characterize the voxel-to-voxel correlation matrix separately for each individual subject and experimental condition. The number of components was set to 40 by default, so that it would be more comparable when using the default ICA SN template.

**Figure 1:**
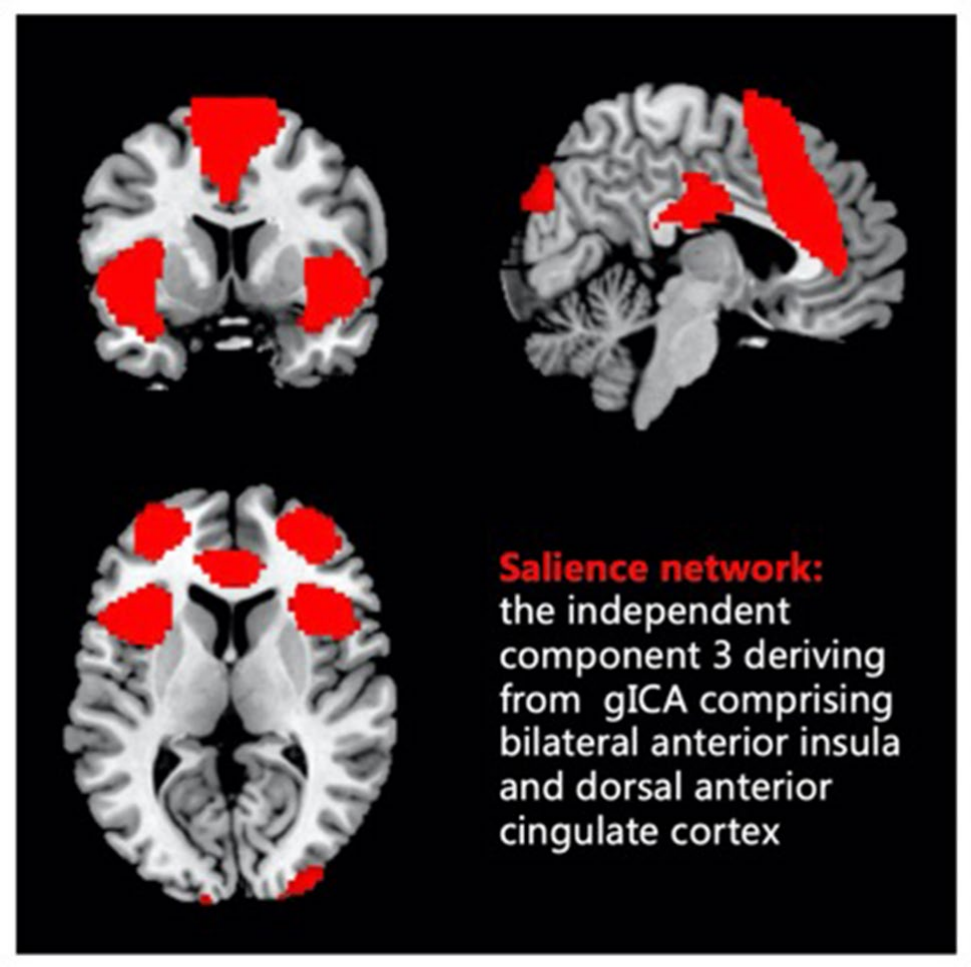
The salience network component identified from group independent component analysis (gICA) of the resting-state fMRI data of all subjects (n = 87).

The correlational spatial match-to-template approach was used in CONN to identify the component representing salience network, which showed the best match to the SN template from human connectome project (HCP, https://www.humanconnectome.org) (r = 0.320). This component includes the main nodes of the SN including bilateral AI and dACC, as well as bilateral rostral prefrontal cortex, and subcortical nodes can also be detected albeit at a weaker level. Spatial distribution of the SN component is highly similar to previous studies^17,25,26^ (Fig. 1). Then this component was defined as the network of interest for the following analyses.

### Functional connectivity analysis

**Figure 2:**
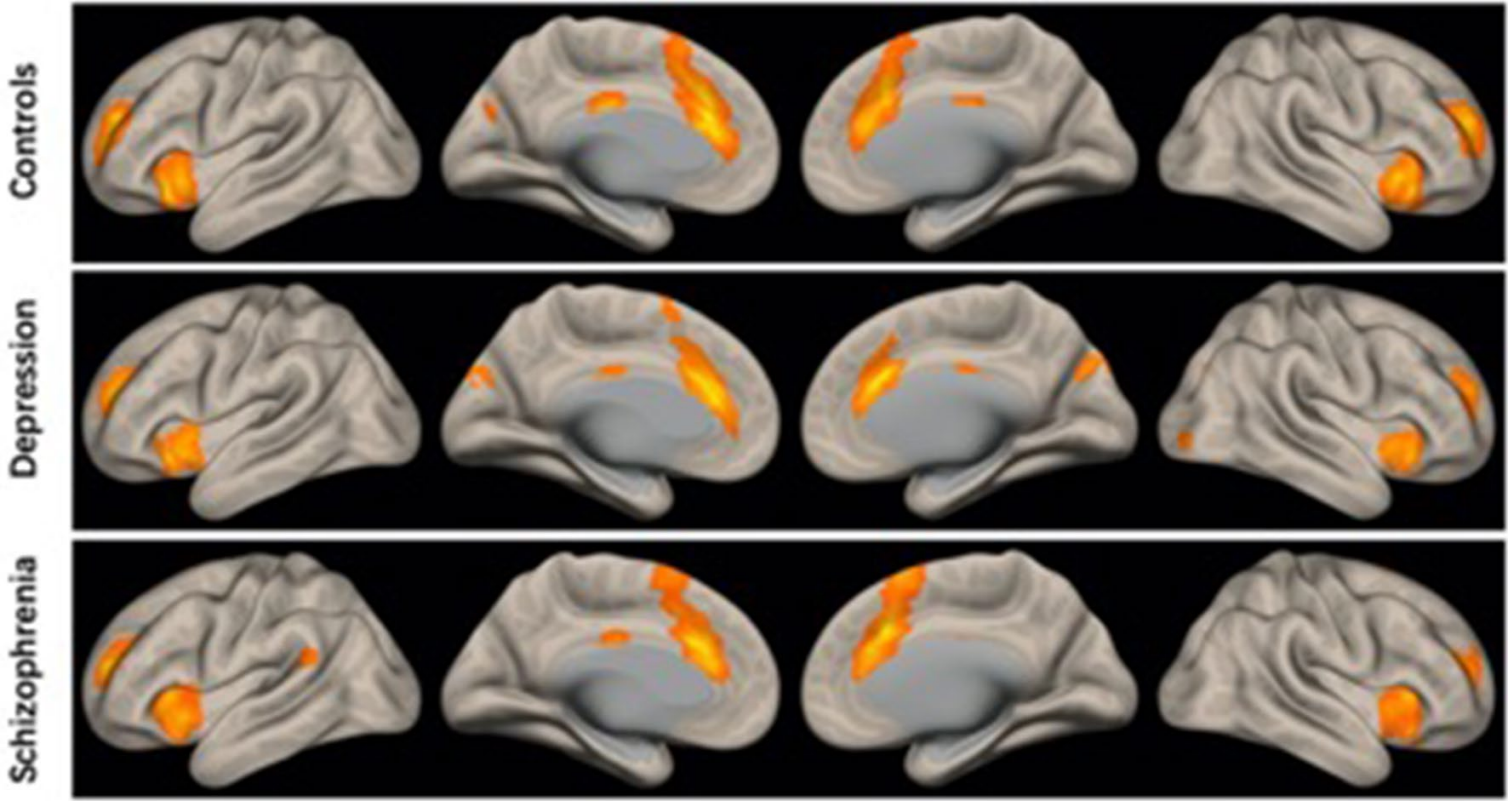
T maps of functional connectivity with the SN and whole brain in the three groups. One-sample t-test was performed for each group (voxel-wise *p* < 0.001 uncorrected and a cluster-wise threshold of *p* < 0.05, FWE-corrected).

Pearson’s correlation coefficients were calculated between the time series of SN component and that of all voxels in the whole brain, a Fisher’s r-to-z transform was applied for increasing normality. The *FC* strength between SN and brain regions was represented by Pearson’s correlation coefficients. The FC maps of SN in each of the three groups were obtained by one-tailed one-sample t-test (voxel-level *p* < 0.05, FWE-corrected), as shown in Fig. 2. To investigate the effect of group and differences between groups, one-way analysis of covariance (ANCOVA) and post-hoc Least Significant Difference (LSD) tests were performed on the FC maps respectively, controlling for age, gender, years of education, and head-motion covariable (FD value). The results for functional connectivity of salience network among groups were displayed at a voxel-wise p < 0.001 uncorrected and a cluster-wise threshold of p < 0.05 FWE-corrected (clusters size > 198).

### Correlation analyses

We further explored the relationships between altered FC of SN and clinical variables. The correlation analyses were performed separately for each patient group. Partial correlations between FC values and illness severity measures (HAMD or PANSS) and duration of illness (DOI) were calculated, controlling for age, gender, years of education, and head-motion covariable (FD value).

## Results

### Demographic and clinical data

Demographic variables across the three groups were compared with the one-way analysis of variance (ANOVA) or independent sample t tests for continuous variables and Chi-square tests for categorical variables. The three groups had no difference in gender, age and education (all p > 0.05). There was also no significant difference in duration of illness between the two patient groups (Detailed information can be seen in Table 1).

**Table 1:** Demographic and clinical characteristic of all participants.

	Healthy controls	Depression	Schizophrenia
	(HC, n = 30)	(DEP, n = 28)	(SCH, n = 29)
Gender (male/female)	11/19	11/17	11/18
Age (years)	28.37 ± 5.08	27.86 ± 6.23	27.48 ± 4.61
Education (years)	14.27 ± 2.16	14.42 ± 2.28	14.21 ± 2.74
Duration of illness (months)	–	25.78 ± 16.75	25.75 ± 18.64
Hamilton depression scale (HAMD)	–	22.89 ± 1.32	–
**Positive and negative syndrome scale (PANSS)**			
Total score	–	–	86.17 ± 9.43
Positive scale score	–	–	23.52 ± 2.97
Negative scale score	–	–	20.27 ± 4.63
General psychopathology	–	–	41.43 ± 5.82
Head motion (framewise displacement)	0.106 ± 0.087	0.132 ± 0.090	0.141 ± 0.103

Groups were matched for gender, age, and education (chi-square test or tone-way ANOVA p > 0.05).

### Functional connectivity of SN

**Figure 3:**
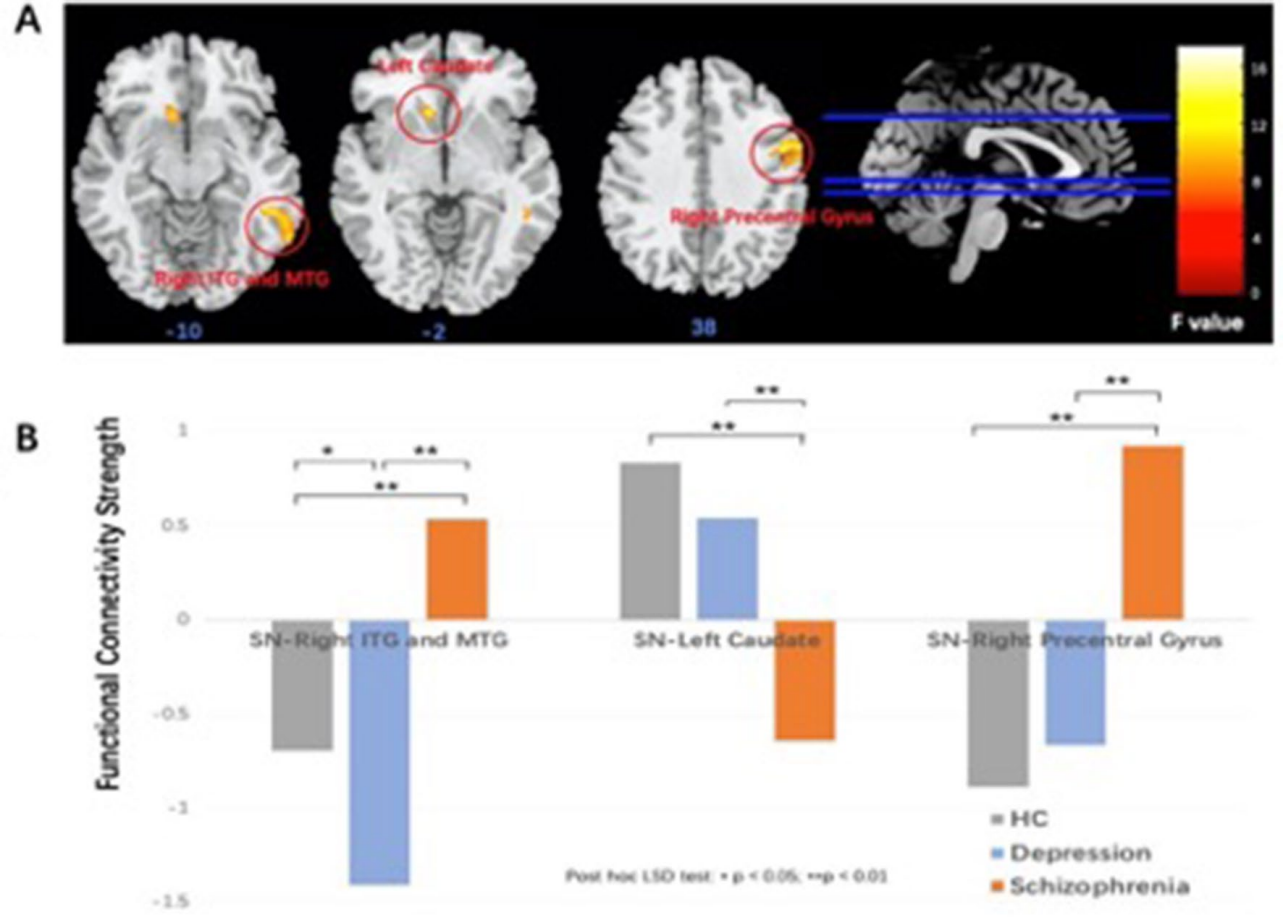
(**A**) Differences of salience network (SN) functional connectivity (FC) in the healthy controls (HC), schizophrenia (SCH), and depression (DEP) by the ANCOVA (voxel-wise *p* < 0.001 uncorrected and a cluster-wise threshold of *p* < 0.05, FWE-corrected), and the colorbar represents F values; (**B**) post-hoc comparisons found that the schizophrenia group showed increased SN-right inferior and middle temporal gyrus (ITG and MTG) FC and SN-right precentral gyrus, as well as increased SN-left caudate in SCH group compared to the other two groups (p < 0.01), the Depression group showed decreased FC SN-right ITG and MTG FC compared to the other two groups (p < 0.05).

The ANCOVA demonstrated a significant group effect in FC with right inferior and middle temporal gyrus (ITG and MTG), left caudate, and right precentral gyrus. Subsequently post-hoc LSD analyses revealed an opposite altered FC pattern between SN and right ITG and MTG for both patient groups (Fig. 3). The DEP group showed a reduced FC between SN and right ITG and MTG compared with HC whereas the SCH group showed an increased FC. In addition, the SCH group showed decreased FC between SN and left caudate, and enhanced FC between SN and right precentral gyrus compared to the other two groups. The results from the ANCOVA and post hoc analyses are presented in Tables 2 and 3 respectively.

**Table 2:** Regions with significant group differences in functional connectivity of salience network in three groups.

Regions	MNI	The z values of FC: Mean (SD)			ANCOVA results		
	Coordinates	HC	DEP	SCH	F value	p value	Cluster size
Right inferior and middle temporal gyrus	50, − 42, − 8	− 0.699 (1.267)	− 1.410 (1.286)	0.536 (1.328)	15.55	1.8 × 10^–6^	211
Left caudate	− 12, 22, − 6	0.833 (1.214)	0.540 (1.142)	− 0.642 (1.030)	13.89	6.1 × 10^–6^	141
Right precentral gyrus	58, 4, 44	− 0.889 (1.254)	− 0.667 (1.281)	0.924 (1.107)	19.26	1.3 × 10^–7^	364

*MNI* Montreal Neurological Institute, *SD* standard deviation, *ANCOVA* analysis of covariance, *HC* healthy controls, *DEP* depression, *SCH* schizophrenia.

**Table 3:** Post-hoc LSD comparisons of FC with significant differences.

FC	Post-poc comparsions p value		
	HC vs DEP	HC vs SCH	DEP vs SCH
SN-Right inferior and middle temporal gyrus	p(HC > DEP) = 0.039	p(SCH > HC) < 0.000	p(SCH > DEP) < 0.000
SN-Left caudate	p(HC > DEP) = 0.587	p(SCH < HC) < 0.000	p(SCH < DEP) < 0.000
SN-Right precentral gyrus	p(HC < DEP) = 0.770	p(SCH > HC) < 0.000	p(SCH > DEP) < 0.000

*SN* salience network, *HC* healthy controls, *DEP* depression, *SCH* schizophrenia, *LSD* least significant difference, *FC* functional connectivity.

### Correlations between abnormal FC and clinical variable

There was no significant correlation between FC strength with significant group effects and clinical variables in the analyses (p > 0.05).

## Discussion

In the current study, we used a data-driven gICA to identify the SN and further investigated the FC of the SN among the patients with schizophrenia and depression by the resting state fMRI. We found that the SCH group and the DEP group showed completely opposing FC between the SN and three regions, including right ITG and MTG, left caudate and right precentral gyrus. In detail, compared with the HC group, the FC between the SN and the right ITG and MTG was enhanced in the SCH group but decreased in the DEP group; the patients with SCH showed decreased FC between the SN and left caudate whereas increased FC between the SN and right precentral gyrus. These findings suggest aberrant resting-state FC pattern of the SN may be a transdiagnostic difference between depression and schizophrenia patients and may play a critical role in the pathogenesis of these two disorders.

The role of the SN in the neural mechanism of psychiatric disorders has been the subject of interest for many researchers, given that the SN is crucial to sustain human emotion and cognition, especially during the detection and processing of salient information^15^. Both schizophrenia and depression are characterized by impairments of cognitive and emotion function. Many SN-related studies have been conducted in schizophrenia, including patients at first episode and chronic phase, as well as individuals at risk for psychosis^25–27^. Dong et al. conducted a meta-analysis of resting-state FC studies in schizophrenia, and they found the SN plays a predominant role in large-scale brain networks, the dysconnectivity of SN may lead to the failure of schizophrenia patients to differentiate self-representation (inner world) and environmental salience processing (outside world)^28^. For the individuals with depression, the number of studies focusing on the SN is relatively small, a meta-analysis study reviewed studies using either seed-based correlation or independent component analysis, and the findings elucidated an increased connectivity between the SN and the anterior DMN^29^.

In this study, we found that compared to the HC group, the FC between the SN and right ITG and MTG was enhanced in SCH group but decreased in the DEP group, suggesting a completely different functional connectivity pattern for the two groups. The patients with schizophrenia showed positive FC whereas the patients with depression showed negative FC. The temporal gyrus plays an important role in the processing of emotion, memory, and mental activities. The ITG and MTG with a significant group effect are overlapped in the temporal part of DMN^30^, which reflects an introspective and spontaneous activity in a non-tasking state and is involved in internal psychological processing and external environmental monitoring^31,32^. Therefore, we suggest that SN-DMN dysconnectivity is significantly associated with both schizophrenia and depression, but in a completely different way. In schizophrenia, the positive FC between the SN and temporal gyrus indicated that the ITG and MTG were coactive with the SN at resting-state, which implies an impaired segregation of task positive regions (SN) and task negative (the ITG and MTG) brain networks. In contrast, negative FC was demonstrated between the SN seed and the temporal gyrus, and is even stronger in depression. The negative FC suggests an anticorrelation, which presumably reflects an active downregulation or inhibition in depression^33^.

Another notable SN FC abnormality observed in the present study suggests a disconnection between SN and subcortical caudate during the resting state in schizophrenia, which is not affected in depression. We found that the SCH group exhibited a negative FC between the SN and left caudate, while the DEP and HC groups exhibited a positive FC. The caudate is located in the rostral striatum and commonly regarded as the subcortical region of SN^34^, which means it should have positive FC with SN, as shown in the healthy controls and patients with depression in our study. However, the SN-caudate in the SCH group shifted from positive to negative FC, suggesting a significant functional disconnection within the SN in schizophrenia, and this dysconnectivity was consistent with our previous study with first episode schizophrenia, which demonstrated that the reduced FC existed within cortico-striatal-thalamic-cortical (CSTC) sub-circuit of SN^26^. For the SN-caudate FC, there was no difference between the healthy subjects and the patients with depression. The relatively intact FC within the SN has also been reported by a recent rsfMRI and ICA study, which explored intrinsic FC within and between resting-state networks (RSNs) in unmedicated patients with depression, they did not find any abnormal intra-network connectivity related to the SN^6^. Therefore, we suppose that the disorganized connectivity within the SN may be only presented in schizophrenia but not depression, since reduced cortico-striatal connectivity was linked with psychotic symptoms, such as hallucinations and delusions^35^. Our findings are in line with previous rs-fMRI evidence, which indicates that SN is increasingly considered as having a fundamental role in the pathophysiology of schizophrenia^27,36–39^.

In addition, this study also found that compared to normal negative FC between SN and precentral gyrus in the DEP and HC groups, the FC was significantly increased and became positive in the SCH group. The precentral gyrus is a key component of sensory and motor movement^40^, and it is believed to play an important role in social cognition, including perception of facial expressions, emotions, and individual desires. A meta-analysis revealed that the engagement level of precentral gyrus in schizophrenia patients was associated with their performance in facial emotion perception^41^. Moreover, both reduced gray matter volume^42^ and local synchrony (regional homogeneity)^43^ have been reported in schizophrenia. The hyperconnectivity between SN and precentral gyrus in schizophrenia means that the information interaction was overactive, which may cause emotional and motional processing dysfunction in schizophrenia.

Taking these results together, the current study supports that the SN FC alterations are more pronounced in the SCH group than in the DEP group. Specifically, we found that the patients with schizophrenia showed opposite coupling between the right temporal gyrus, left caudate, and right precentral gyrus and the SN, while the patients with depression exhibited original negative connectivity mode between the temporal gyrus and the SN. In addition, the findings of current study are in agreement with the previous, arguing that FC features of SN may play an important role in distinguishing schizophrenia from depression^7^. However, we failed to confirm any relationships between the symptom’s severity and disease’s duration in the patients as previous studies^44–46^, which may be due to the relatively small sample size in each group and the high heterogeneity in both diseases.

## Conclusions

In summary, our study provides evidence for different FC alterations of the SN in schizophrenia and depression. The findings suggest that the resting-state FC of SN may be a transdiagnostic difference between depression and schizophrenia patients and may play a critical role in the pathogenesis of these two disorders.

### Limitation

Several limitations should be considered in this study. First, the sample size was relatively small in each group, which may have yielded unreliable results, a larger number of participant samples is needed to increase the reliability and sensitivity. Second, most patients were taking medication, so the systematically different medications for schizophrenia and depression may have different effects on functional connectivity. This source of variance may have acted as a major confound in transdiagnostic analyses. Third, the study was a cross-sectional design and all the patients were at their acute phase, so it remains unclear when the FC abnormalities of FC emerged and how they change as the disease progresses and longitudinal follow-up studies are needed.
